# Viscotaxis: An Emerging Driver for Directed Cell Migration

**DOI:** 10.3390/cells15171531

**Published:** 2026-08-25

**Authors:** Zijian Chen, Zhen Wang, Guanglin Wang

**Affiliations:** 1 Trauma Medical Center, Department of Orthopaedic Surgery, West China Hospital, Sichuan University, Chengdu 610041, China; 2024324025141@stu.scu.edu.cn (Z.C.); wangzhen_tmc@stu.scu.edu.cn (Z.W.); 2 Department of Orthopaedic, West China Hospital, Sichuan University, Chengdu 610041, China

**Keywords:** directed cell migration, cell mechanics, viscotaxis, viscoelasticity

## Abstract

Directed cell migration orchestrates embryonic development, wound repair, immune surveillance and malignant tumor invasion. Viscotaxis—directed cell migration along spatial gradients of extracellular fluid viscosity or matrix loss modulus—has emerged as a candidate mechanotaxis modality that may guide cells through heterogeneous viscoelastic tissues; however, its biological functions and molecular underpinnings remain largely uncharacterized, and direct evidence in mammalian systems is still limited to a small number of studies. This mini-review first defines viscotaxis and distinguishes it from the related but physically different mechanical quantities with which it is frequently conflated—substrate stiffness, matrix viscoelasticity, and stress relaxation—and then summarizes its physiological and pathological relevance across development, tissue homeostasis, immunity, and cancer. We outline the putative multi-step mechanosensory cascade governing viscotactic responses, examine how viscotaxis may synergize or compete with durotaxis, haptotaxis, chemotaxis, electrotaxis, and phototaxis under mixed microenvironmental cues, and compare the distinct hydrodynamic and steric-exclusion mechanisms proposed across spiral microbes, flagellated eukaryotes, mammalian cells, and embryonic tissues. Throughout, we explicitly distinguish evidence obtained under viscosity gradients from that obtained under uniformly elevated viscosity and established findings from working hypotheses. Finally, we discuss current methodological bottlenecks and unresolved conceptual debates and propose biomaterial tools and therapeutic strategies to advance viscotaxis from a biophysical curiosity toward a core principle of cellular mechanobiology.

## 1. Introduction

Directional cell migration, broadly termed taxis, enables cells to navigate heterogeneous microenvironments and governs embryonic development, wound repair, immune surveillance, and functional biomaterial design [1]. In native tissues, cells experience not only chemical cues but also a mechanically heterogeneous environment: the fluids that bathe cells vary in viscosity, and the extracellular matrix varies in stiffness and viscoelasticity. Intuitively, viscotaxis describes the tendency of cells and microorganisms to migrate directionally along gradients of these physical properties—formally, it is defined as directed cell migration in response to a spatial gradient of extracellular fluid viscosity or of the matrix loss modulus, the viscous, energy-dissipating component of a viscoelastic substrate. Unlike chemotaxis, which follows gradients of soluble chemical ligands, viscotaxis is guided by purely physical gradients; it is therefore a putative mechanotaxis modality whose biological functions and molecular underpinnings remain largely uncharacterized, with direct evidence in mammalian systems currently limited to a small number of studies [2].

The concept was first introduced in 1978, when Petrino and Doetsch observed that *Leptospira interrogans* preferentially migrated from non-viscous environments into regions of higher viscosity, a positive behavioral response they termed “viscotaxis” that was proportional to the steepness of the viscosity gradient and reproducible with inert polymers, thereby excluding chemotactic artifacts [3]. Despite this early description, the phenomenon remained largely dormant for decades, overshadowed by the ascendancy of chemotaxis and, more recently, durotaxis as the dominant paradigms of directed cell migration. With advances in engineered viscoelastic biomaterials and high-resolution live-cell imaging, viscotaxis has re-emerged as a candidate mode of mechanical guidance that may operate across scales, from bacterial microswimmers and flagellated eukaryotes to mammalian cells and embryonic tissues, although direct evidence in higher organisms remains sparse. However, the field currently faces fundamental gaps: the molecular identity of the primary viscosity sensor remains elusive, the extent to which viscotaxis operates independently of or synergistically with coexisting stiffness and adhesion ligand gradients is debated, and direct in vivo validation in native tissue microenvironments is lacking. Addressing these unresolved questions will be essential to establish viscotaxis not merely as a biophysical curiosity but as a core principle of mechanobiology with implications for development, tissue homeostasis, and disease.

Early work primarily documented phenotypic bacterial accumulation in viscous media without resolving the underlying cellular sensing cascades or the crosstalk with coexisting physical and biochemical directional cues, leaving critical biological questions unaddressed. The mechanistic and translational exploration of viscotaxis across eukaryotic cells and complex tissues has only flourished within the past decade. To bridge this historical and mechanistic gap and build upon recent cross-scale evidence that collectively points toward the existence of viscosity-guided directional responses, this mini-review provides a preliminary systematic synthesis of viscotaxis as a candidate mode of directed cell migration driven by spatial gradients of extracellular fluid viscosity or matrix loss modulus, while acknowledging that the field remains in its early stages. Grounded in first-principles conceptual frameworks, we delineate the biophysical rationale for viscotaxis and elaborate on its underlying regulatory cascades, including the mechanotransductive axis and its synergistic or antagonistic interactions with other taxis modalities, such as durotaxis, haptotaxis, chemotaxis, electrotaxis and phototaxis. By reconciling contemporary multi-tissue mechanistic findings, ranging from spiral prokaryotes and flagellated microswimmers to mammalian cells and embryonic tissues, this review advances a holistic, cross-scale understanding of cellular mechanosensing and mechanotransduction.

## 2. Definition and Biological Relevance of Viscotaxis

### 2.1. Distinguishing Fluid Viscosity, Matrix Viscoelasticity, and Viscotaxis

Extracellular fluid viscosity and matrix viscoelasticity are related but physically and experimentally distinct quantities, and we therefore define them explicitly at the outset. Extracellular fluid viscosity is a property of the liquid phase surrounding cells—interstitial fluid, mucus, blood plasma, or culture medium—and quantifies the resistance of the fluid to flow; it can be raised uniformly, for example, by adding inert thickening polymers, or vary spatially to form a viscosity gradient. Matrix viscoelasticity, in contrast, is a property of the solid extracellular matrix: a viscoelastic network both stores elastic energy, characterized by the elastic (storage) modulus, and dissipates energy, quantified by the loss modulus or, equivalently, by the stress-relaxation time over which the matrix relaxes an imposed stress. The viscosity of the fluid bathing a cell and the loss modulus of the matrix to which it adheres are therefore not interchangeable, and they are sensed through potentially distinct mechanisms: fluid viscosity acts through viscous drag on the cell surface, whereas loss modulus is engaged through adhesion-mediated force transmission to the matrix.

Throughout this review, we use the term viscotaxis to denote directed migration along a spatial gradient of either extracellular fluid viscosity or matrix loss modulus, the two forms in which the phenomenon has been reported [2,3], and we explicitly distinguish this gradient-driven behavior from the cellular response to uniform viscosity elevation, which alters cell mechanics and signaling but provides no directional cue. We also organize the cited evidence according to the physical variable examined—fluid-viscosity gradients, loss-modulus gradients in stiffness-matched matrices, uniform viscosity elevation, or matrix stress relaxation—and where evidence from uniform-viscosity experiments is used to infer mechanisms of gradient-driven migration, we present the inference as a working hypothesis rather than an established finding. Table 1 summarizes these definitions and organizes the principal evidence cited in this review according to the physical variable examined.

**Table 1:** Terminology used in this review and organization of the cited evidence according to the physical variable examined.

Physical Quantity	Operational Definition and Experimental Realization	Representative Evidence Cited in This Review
Extracellular fluid viscosity (uniform elevation)	Inert thickening polymers added to the culture medium; elevated interstitial fluid viscosity in tumors	Membrane ruffling and lamellipodial load adaptation [4,5]; liver cancer mechanosensing [6]; ARP2/3–NHE1–TRPV4–RhoA/ROCK cascade and cancer dissemination [7]; glioblastoma mechanical priming [8]; stem-cell fate and mechanical memory [9]
Extracellular fluid viscosity gradient	Spatial viscosity variation in mucus layers, interstitial fluids, or microfluidic devices	Leptospira viscotaxis [3]; intestinal mucus gradients [10]; microswimmer and flagellate hydrodynamic theory [11,12,13]; Brachyspira mucin attraction [14]; microalgal viscophobic responses [15,16]
Matrix loss modulus (spatial gradient)	Stiffness-matched viscoelastic hydrogels presenting a graded loss modulus	Mesenchymal stem-cell viscotaxis [2]; coupled elastic–viscous gradient models [17,18]; microfluidic and phototunable viscoelastic hydrogels [19,20]
Matrix stress relaxation	Time-dependent decay of an imposed stress in a viscoelastic substrate	Stress-relaxation-enhanced cell spreading and migration [21]; viscoelastic materials for cell engineering [22]

### 2.2. Physiological and Pathological Significance

Spatial gradients of viscosity and loss modulus arise naturally in many physiological niches. In the intestine, the mucus layer exhibits both vertical (lumen-to-epithelium) and longitudinal (proximal-to-distal) viscosity gradients that shape the mucosal barrier and the spatial organization of the resident microbiota [10], and pathogenic spirochetes exploit these gradients, migrating toward high-viscosity mucin to colonize the mucosal niche [14]. During embryonic development, glycosaminoglycan-rich matrices establish viscosity-dependent environments that have been proposed to guide cell and neurite migration and to pattern morphogenesis [23]. Interstitial fluid viscosity further increases with macromolecular crowding, and blood and lymphatic fluids impose viscous loads on circulating and extravasating cells [24].

In disease, viscosity dysregulation is most extensively documented in cancer. Solid tumors exhibit elevated interstitial fluid viscosity owing to macromolecular crowding, impaired lymphatic drainage, and extracellular matrix degradation [24], and elevated viscosity enhances the migration and dissemination of mammary tumor cells and the mechanosensing and migration of liver cancer cells [6,7]. In glioblastoma, increased tissue fluidity and interstitial viscosity are associated with tumor progression and with the mechanical priming of malignant cells for invasion [8,25]. Beyond motility, extracellular viscosity also regulates cell fate and function: in human mesenchymal stem cells, elevated fluid viscosity biases differentiation toward an osteogenic lineage, imprints a durable osteogenic mechanical memory, and promotes an immunosuppressive phenotype that favors M2 macrophage polarization—effects observed across substrates of different stiffness and stress-relaxation times [9]. These findings establish viscosity sensing as a regulator of tissue homeostasis, immunity, and regeneration in addition to directed migration, and they motivate the study of viscotaxis as a potential guidance mechanism in development, inflammation, and metastasis, as well as a target for biomaterial and therapeutic design.

## 3. Viscotaxis Cascade Mechanism

**Figure 1:**
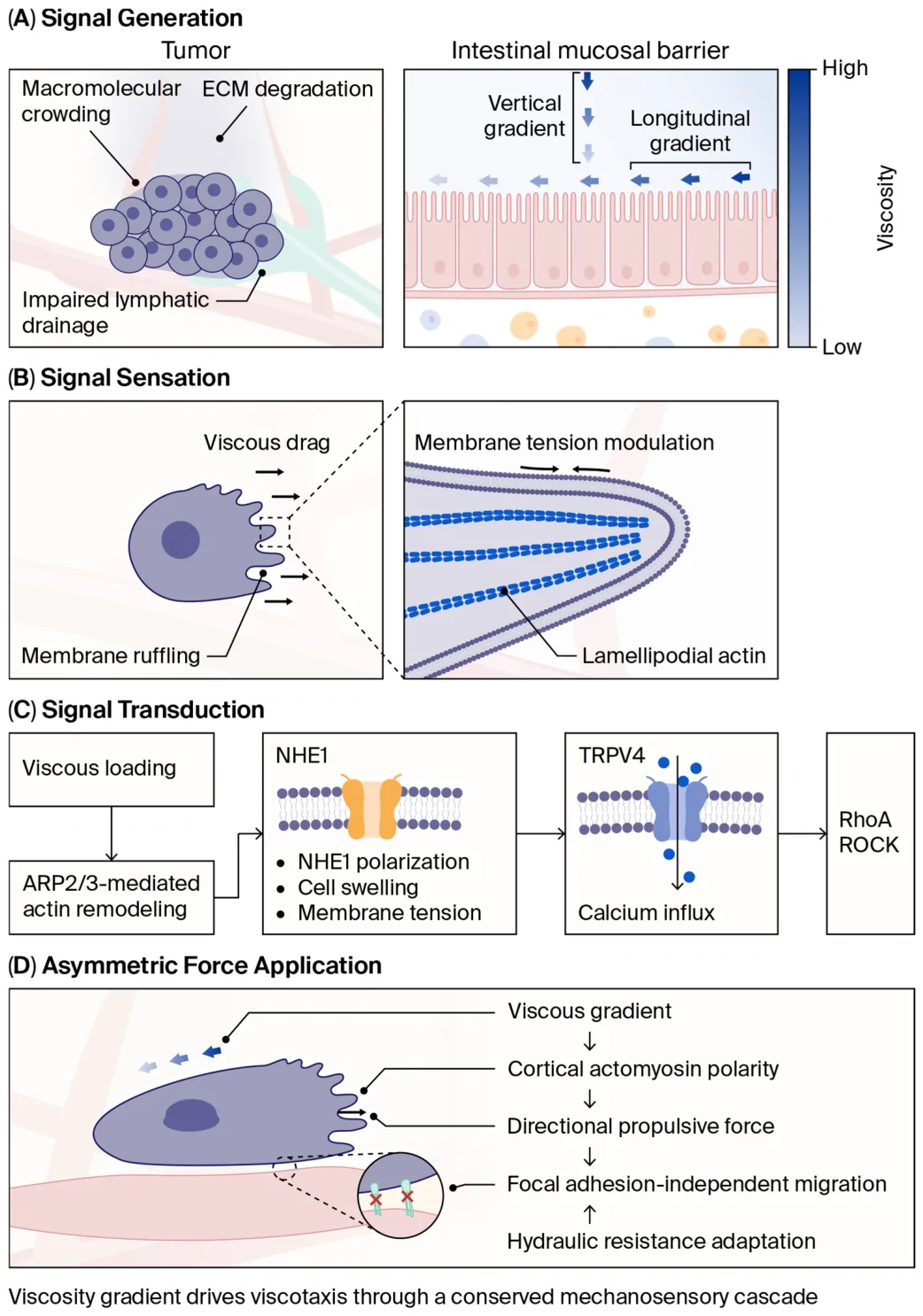
Working model of the multi-step mechanosensory cascade proposed to govern viscotaxis, integrating mechanisms identified in viscosity-gradient studies with those characterized under uniformly elevated extracellular viscosity [2,7]. (**A**) Signal generation: stable gradients of extracellular fluid viscosity arise in pathophysiological niches—for example, in tumors through macromolecular crowding, extracellular matrix degradation, and impaired lymphatic drainage—and in the intestinal mucosal barrier as vertical (lumen-to-epithelium) and longitudinal (proximal-to-distal) gradients within the mucus layer [10,24]. Background color washes indicate tissue microenvironments, and the blue gradient scale bar denotes viscosity from low (light) to high (dark). (**B**) Signal sensation: membrane ruffling serves as a dedicated mechanosensor of extracellular fluid viscosity [5], and lamellipodial actin networks modulate membrane tension in response to mechanical load [4]. (**C**) Signal transduction: viscous loading triggers ARP2/3-mediated actin remodeling, which drives Na^+^/H^+^ exchanger 1 (NHE1) polarization and cell swelling, thereby elevating membrane tension and activating the mechanosensitive channel TRPV4; the resulting calcium influx engages the RhoA/ROCK pathway to establish global cell polarity. This step has so far been characterized mainly under uniform viscosity elevation [7]. (**D**) Asymmetric force application: the polarity signal reorganizes cortical actomyosin to generate directional propulsive force; in confined microenvironments, migration can proceed independently of focal adhesions, aided by hydraulic-resistance adaptation [27,28]. Red crosses mark the absence of focal adhesions during confinement-associated, adhesion-independent migration. (**C**,**D**) incorporate concepts developed from subsequent uniform-viscosity and confinement studies and remain to be fully validated under gradient conditions.

To identify the universal principles underlying directed cell migration, a conceptual framework has been proposed that organizes all forms of cell taxis into four core pillars: signal generation, sensation, transduction, and asymmetric force application [1]. Figure 1 applies this framework to viscotaxis, integrating mechanisms proposed in the original viscosity-gradient studies with those characterized subsequently under uniformly elevated extracellular viscosity [2,7]. In this working model, the viscosity gradient itself constitutes the directional signal (Figure 1A); the signal is sensed at the cell surface through membrane ruffling and lamellipodial actin dynamics (Figure 1B); it is transduced into intracellular polarity through the ARP2/3–NHE1–TRPV4–RhoA/ROCK signaling axis (Figure 1C); and it is ultimately converted into spatially asymmetric propulsive forces that drive directed migration (Figure 1D). Because the molecular evidence for individual steps has been obtained largely under uniform viscosity elevation rather than true gradients, we explicitly indicate below which steps are established and which remain inferred.

As introduced in Section 2.2 and illustrated in Figure 1A, stable viscosity gradients serve as endogenous physical cues across tissue microenvironments, ranging from the tumor interstitium [24] to the intestinal mucus layer [10]. At the sensing stage, cells detect viscous drag independently of canonical ligand–receptor interactions: membrane ruffling acts as a dedicated mechanosensor of extracellular fluid viscosity [5], lamellipodial actin networks adapt to mechanical load by altering membrane tension [4], and the resulting viscosity-induced mechanosensing phenotype has been independently validated in liver cancer cells [6]. During signal transduction, the physical cue is converted and amplified into global polarity signals through a conserved cascade. This cascade has been most extensively characterized in the context of uniformly elevated extracellular viscosity, where viscous loading triggers actin-related protein 2/3 complex (ARP2/3)-mediated actin remodeling that drives Na^+^/H^+^ exchanger 1 (NHE1) polarization and cell swelling, thereby enhancing membrane tension and activating the mechanosensitive ion channel transient receptor potential vanilloid 4 (TRPV4); the resulting calcium influx engages the RhoA/Rho-associated coiled-coil-containing protein kinase (ROCK) pathway to propagate and amplify the signal [7]. The same core axis, extended by integrin-linked kinase-dependent cell spreading and YAP-dependent transcriptional outputs, also couples viscosity sensing to stem-cell fate specification [9]. Whether this cascade is engaged by spatial viscosity gradients—and whether it is sufficient to generate directional migration—remains to be tested directly, and additional mechanosensors, including PIEZO1 and the volume-regulated anion channel SWELL1, may act in parallel with, or redundantly to, TRPV4 [26].

At the execution stage, the polarity signal is converted into spatially asymmetric propulsive forces. Importantly, the force-generating machinery appears to be context-dependent. On two-dimensional adhesive substrates, where viscotaxis was first validated in mesenchymal stem cells migrating along loss-modulus gradients, migration relies on the same adhesion-mediated force-transmission machinery as canonical durotaxis [2]. In confined three-dimensional microenvironments, by contrast, cells can migrate along viscosity gradients independently of focal adhesions: hydraulic resistance induces adaptive phenotypic transitions that optimize force output and sustain adhesion-independent migration [27,28], and confinement additionally engages nuclear deformation, adhesion-independent force transmission, and metabolic reprogramming as integral components of the migratory response [26]. These two modes are not mutually exclusive—cells may switch between adhesion-dependent and adhesion-independent viscotaxis according to matrix architecture and confinement—although the molecular switch remains unresolved. Overall, these findings, drawn largely from studies on uniform viscosity elevation and from theoretical considerations, suggest a working model in which viscosity gradients may trigger a viscotaxis cascade (Figure 1). This model provides a preliminary framework for understanding how cells could respond to loss-modulus gradients, but it is important to emphasize that several steps remain speculative; in particular, direct demonstration of this cascade operating as a bona fide taxis mechanism under gradient conditions and in diverse cell types has not yet been achieved.

## 4. Crosstalk Between Viscotaxis and Other Taxis Modalities

While viscotaxis shares conceptual overlaps with other canonical taxis modalities, it relies on distinct mechanotransduction machinery, and their crosstalk remains incompletely understood. This section compares viscotaxis with five established taxis modalities—durotaxis, haptotaxis, chemotaxis, electrotaxis, and phototaxis—each defined at the beginning of the corresponding subsection. For each modality, we focus on the biological systems in which interplay with viscosity gradients has been observed or proposed, and we explicitly distinguish experimental evidence from theoretical prediction and from untested hypothesis.

### 4.1. Durotaxis

Durotaxis refers to directional migration guided by gradients of substrate stiffness (elastic modulus) and represents the most extensively characterized mechanotaxis paradigm to date. Because native tissues are viscoelastic, gradients of elastic modulus frequently coexist with spatial variations in matrix loss modulus and of pericellular fluid viscosity; these three quantities are physically distinct and engage different cellular machinery. Unless stated otherwise, the term “viscosity gradient” in this subsection refers specifically to a spatial gradient of the matrix loss modulus. Durotaxis and viscotaxis are thus conceptually related but mechanistically distinct taxis modalities whose interplay remains incompletely understood [1,2].

Viscotaxis was first experimentally validated in mesenchymal stem cells cultured on stiffness-matched hydrogels presenting a graded loss modulus, in which migration relied on the same adhesion-mediated mechanotransduction pathway as canonical durotaxis [2]. Subsequent studies decoupled matrix stiffness from stress relaxation, revealing that viscous dissipation synergizes with stiffness to amplify directed migration—for example, enhanced substrate stress relaxation promotes filopodia-mediated cell spreading and migration independently of the elastic modulus [21]. The physical basis of this coupling is captured by the motor–clutch paradigm, in which myosin-driven retrograde actin flow is coupled to the substrate through focal-adhesion “clutches”: spatially uneven cell–matrix friction biases traction and thereby drives durotaxis, and the same friction asymmetry can produce positive durotaxis (migration toward stiffer regions, when clutch engagement strengthens with stiffness), negative durotaxis (migration toward softer regions, when clutch or motor dynamics saturate), or controlled migration, depending on clutch engagement and substrate properties [29]. Representative examples of this interplay include mesenchymal stem cells migrating up loss-modulus gradients on stiffness-matched hydrogels; cells on viscoelastic substrates in which stiffness and loss-modulus gradients jointly polarize adhesion turnover and actomyosin contractility, with synergistic or antagonistic outcomes depending on gradient alignment [17]; and quantitative models predicting that, when the two gradients oppose each other, durotaxis dominates directionality whereas opposing loss-modulus gradients weaken or even reverse locomotion [18]. These predictions are supported experimentally: microfluidic platforms generating coupled stiffness and stress-relaxation gradients reveal lineage-dependent migratory preferences [19], and phototunable hydrogels with spatiotemporally adjustable viscoelastic gradients recapitulate these migratory phenotypes in invasive tumor cells [20]. Collectively, durotaxis and viscotaxis are intrinsically interconnected in governing directed cell migration: as mechanistically linked mechanotaxis subtypes, they share adhesion-dependent force-transduction pathways and produce synergistic or antagonistic outcomes depending on gradient alignment, with durotaxis typically determining migration directionality and loss-modulus gradients modulating the strength and plasticity of the directional response.

### 4.2. Haptotaxis

Haptotaxis is directed cell migration in response to gradients of immobilized adhesion ligands—extracellular matrix components such as fibronectin, collagen, and laminin that are bound to the substrate—and represents a fundamental mechanism by which cells interpret spatial variations in substrate-bound biochemical cues. At the molecular level, haptotactic sensing is mediated by cell-adhesion molecules, principally integrins—for example, α5β1 and αvβ3 integrins binding fibronectin, α1β1 and α2β1 integrins binding collagen, and α6β1 integrin binding laminin—which cluster into focal adhesions containing talin, vinculin, paxillin, and focal adhesion kinase to transduce ligand-density differences into traction forces, with further modulation by cell-surface proteoglycans and other adhesion receptors [23,30]. Adhesive-ligand and viscosity gradients often coexist across tissue matrices and interstitial spaces, and because all forms of directed cell migration rely on shared cytoskeletal and force-generating machineries, spatial variations in extracellular viscosity—whether of the matrix or of the surrounding fluid—can intersect with haptotactic signaling pathways to shape migratory outputs.

Mechanistically, matrix viscosity is expected to modulate haptotaxis by shaping the core machinery of adhesive-gradient sensing: viscous loading alters focal-adhesion turnover kinetics and the subcellular distribution of traction forces, thereby influencing the magnitude of cellular responses to immobilized adhesive-ligand gradients [22]. Consistently, a multiscale whole-cell theoretical framework predicts that the spatial coupling of matrix-viscosity and adhesion gradients tunes haptotactic sensitivity and can even reverse migration direction by rewiring intracellular polarity signaling and the subcellular force balance [17]. It should be emphasized, however, that direct experimental tests of viscotaxis–haptotaxis crosstalk under defined viscosity gradients are currently lacking: the supporting literature consists of theoretical models and of studies on viscoelastic materials, and this relationship should therefore be regarded as a mechanistic prediction awaiting primary experimental validation. If confirmed, such crosstalk would position viscosity gradients as contextual regulators of haptotaxis and provide a biophysical basis for interpreting heterogeneous cell-invasion patterns in complex tissue microenvironments, where adhesive and viscous properties vary spatially.

### 4.3. Chemotaxis

Chemotaxis, directed cell migration in response to gradients of soluble chemoattractants, represents the most intensively studied and historically dominant taxis paradigm. The interplay between physical and biochemical signals is increasingly recognized as a primary determinant of chemotactic performance, affecting the efficiency, directional stability, and long-term persistence of cellular migration within heterogeneous tissue microenvironments [30]. Within this context, gradients of extracellular fluid viscosity may intersect with chemotaxis at multiple levels: at the extracellular scale, through viscosity-dependent modulation of ligand diffusion kinetics; and at the intracellular scale, through mechanical priming of cellular responsiveness to chemoattractant signals.

In complex tissue microenvironments ranging from the tumor stroma to intestinal mucus layers, spatial gradients of extracellular fluid viscosity and soluble chemoattractants frequently co-occur, forming an integrated signaling network that shapes migratory outputs. At the extracellular level, this coupling is captured quantitatively by the Stokes–Einstein relation, $D=\frac{k_{B}T}{6\pi \eta r}$, which states that the diffusion coefficient *D* of a soluble ligand of hydrodynamic radius *r* is inversely proportional to the medium viscosity *η* at absolute temperature *T* [31]. Spatial variations in viscosity therefore generate heterogeneous ligand-diffusion kinetics: chemoattractant molecules diffuse more slowly within high-viscosity regions, producing local ligand retention, steepened gradient slopes, and remodeled steady-state concentration profiles relative to uniformly low-viscosity conditions. This physical reshaping enhances the directionality and persistence of chemotactic cues, providing sharper spatial guidance for cell migration.

Beyond remodeling the extracellular cue landscape, viscosity gradients act as mechanical conditioning signals that prime cell-intrinsic chemotactic responsiveness: sustained exposure to viscous stress induces cytoskeletal remodeling, nuclear translocation of the mechanotranscriptional regulator Yes-associated protein (YAP), and transcriptional upregulation of mitochondrial energy metabolism, establishing a stable pro-invasive cellular state that enhances both migration speed and directional fidelity toward chemoattractant gradients [7]. In pathophysiological contexts such as the glioblastoma invasive margin, this dual regulation is expected to form a functional synergy, in which increased interstitial viscosity traps signaling molecules to form robust chemoattractant fields while priming malignant cells for enhanced chemotactic invasion, collectively driving progressive tissue infiltration [8,25]. Collectively, these findings establish viscosity gradients as important contextual regulators of chemotaxis, bridging extracellular biophysical properties with the spatial organization of guidance signals and the migratory competence of target cells, thereby adding a physical dimension to the canonical understanding of directed cell migration.

### 4.4. Electrotaxis

Electrotaxis refers to directional cell migration guided by endogenous or applied electric fields, a process that operates through electrokinetic mechanisms distinct from those of viscotaxis. At present, there is no direct experimental evidence that viscosity gradients modulate cellular electrotaxis; the proposed connection between the two modalities instead rests on physicochemical studies of passive ion transport, which we summarize here before discussing its possible biological implications as a hypothesis. Theoretical work on viscomigration predicts that, through the Stokes–Einstein relation, a spatial viscosity gradient modulates local ion diffusivity and drives a net drift of ions and colloids toward lower-viscosity regions, extending viscosity-guided movement from active microswimmers to passive particle transport [32]. This prediction is supported by nanofluidic experiments on viscophoresis, in which controlled viscosity gradients established along glass nanochannels generated measurable ionic currents arising from net counterion drift toward low-viscosity zones, demonstrating that viscosity gradients alone can drive net ionic migration without an external electric field and can act additively with applied fields [33]; subsequent modeling has refined the quantitative description of this coupling [34]. Because endogenous electric fields and viscosity gradients coexist in vivo—for example, at wounded epithelia, where transepithelial potentials guide the electrotactic migration of keratinocytes and fibroblasts while wound exudate and macromolecular crowding establish local viscosity gradients—viscosity-driven ion drift could, in principle, locally reshape ionic distributions and electric-field profiles and thereby modulate cellular electrotaxis. We emphasize, however, that this physicochemical coupling has so far been demonstrated only in abiotic nanochannel systems; whether it influences the electrotactic migration of cells in biological microenvironments remains an untested hypothesis, which could be examined directly by combining defined viscosity gradients with applied electric fields in microfluidic cell-migration assays.

### 4.5. Phototaxis

Phototaxis, directed migration in response to light gradients, represents the dominant navigational strategy in many flagellated microalgae, enabling these organisms to optimize photosynthetic efficiency. Phototaxis and viscotaxis, as two core physicotactic modalities coexisting in aquatic microenvironments, jointly shape the directional navigation of flagellated microalgae such as *Chlamydomonas reinhardtii* and *Volvox carteri* [35]. Both modalities rely on coordinated flagellar beating dynamics, creating a shared motor machinery through which viscosity gradients can compete with or modulate phototactic steering.

Viscosity gradients alter phototactic fidelity indirectly by modulating flagellar rotation and swimming kinematics: elevated medium viscosity suppresses colony spinning speed, thereby impairing the light-sampling cycle and weakening adaptive phototactic performance, as validated in Volvox assays [36]. Meanwhile, inherent viscophobic turning behavior redirects algal trajectories toward low-viscosity zones, creating hydrodynamic bias that competes or cooperates with light-guided locomotion; sharp viscosity interfaces further trigger scattering reorientation to disrupt phototactic alignment [15,16]. Mechanistically, both phototactic steering and viscotactic reorientation rely on coordinated flagellar beating dynamics, forming shared downstream motor machinery that integrates optical and viscous physical cues to determine net migration trajectories. For synthetic light-driven microswimmers, this coupling implies that light intensity gradients can be rationally tuned to counteract or amplify the directional bias imposed by spatial viscosity heterogeneities, enabling programmable navigation when multiple physical fields coexist.

## 5. Cross-Scale Mechanisms of Viscotaxis

Directional viscotaxis in response to viscosity or loss-modulus gradients has been proposed as a fundamental mechanism by which cells perceive microenvironmental mechanical cues and undergo oriented migration, although direct evidence across diverse cell types and in vivo contexts remains limited. This section synthesizes the underlying principles from the perspective of the responding biological systems—moving from spiral prokaryotes and active microswimmers to flagellated eukaryotic cells, mucosal pathogens, and embryonic tissues—and indicates, for each system, whether the supporting evidence is theoretical or experimental.

### 5.1. Geometry-Driven Viscotaxis in Spiral Prokaryotes

Pioneering theoretical studies on spiral-shaped prokaryotes have established the hydrodynamic mechanism underlying bacterial viscotaxis. Non-uniaxial cell geometries generate asymmetric viscous drag across the cell body, driving directional movement toward higher-viscosity regions, and account for the viscotactic phenotypes of *Spiroplasma* and *Leptospira* species [11]. Notably, this paradigm implies that viscosity-directed migration can arise solely from geometric asymmetry, without any active sensing or signaling. In essence, the cell body functions as a physical transducer that converts the scalar viscosity field into a directional mechanical cue. This geometry-driven mechanism stands in marked contrast to conventional chemotaxis that relies on dedicated receptor arrays and intracellular signal transduction cascades, highlighting a fundamental divergence in the physical principles underlying different modes of taxis.

### 5.2. Viscotactic Behaviors of Axisymmetric Active Microswimmers

Extending this framework to spherical microswimmers, follow-up theoretical studies revealed that even axisymmetric active particles exhibit negative viscotaxis toward lower-viscosity environments. Their migration trajectories and gradient sensitivity are determined by their propulsion gait (pusher, puller, or neutral swimmer), broadening our understanding of how cell shape and locomotion mode shape viscotactic responses [12]. This finding is conceptually significant because it demonstrates that viscotaxis is not limited to spiral or elongated geometries. Instead, any active particle that generates a hydrodynamic flow field can exhibit viscosity-directed migration, provided that its propulsion mechanism produces a force dipole whose orientation and magnitude vary with the local viscosity. The distinction between pusher- and puller-type swimmers further indicates that the direction of viscotaxis, whether positive or negative, is not an intrinsic property of the medium but emerges from the interaction between the swimmer’s mechanical activity and viscous environment. Together, these results suggest that viscotaxis may be a general property of self-propelled particles in viscous gradients.

### 5.3. Flagellum-Mediated Positive Viscotaxis in Eukaryotic Flagellated Cells

In eukaryotic flagellated cells, a distinct mechanism enabling robust positive viscotaxis has been identified through theoretical modeling of flagellar hydrodynamics. Active flagellar beating generates transverse drag imbalances across viscosity gradients to power steady upward-viscosity migration. The strength of this directional response scales with the fourth power of beat amplitude and is further modulated by flagellar flexibility [13]. This fourth-power scaling is particularly revealing, as it indicates that the evolved flagellar machinery of eukaryotic cells achieves a level of viscotactic sensitivity that greatly exceeds what passive hydrodynamic asymmetry alone can provide. Additionally, the strong dependence on beat amplitude indicates that cells can actively tune their viscotactic response by modulating the flagellar waveform, offering a direct association between molecular motor activity and directional sensing. Moreover, incorporating flagellar flexibility into the theoretical framework highlights a recurring theme in mechanobiology, that structural compliance is not merely a passive material property but an active determinant of how cells interpret and respond to mechanical signals.

### 5.4. Physiological Viscotaxis of Mucosal Spirochetes During Niche Colonization

Moving from mechanistic dissection to physiological validation, experimental research on the intestinal spirochete *Brachyspira pilosicoli* confirmed the functional relevance of viscotaxis in mucosal colonization. The bacteria migrate toward high-viscosity mucin that matches the physiological viscosity of colonic mucus. Consistently, the motility of *B. pilosicoli* is enhanced in viscous environments [37]. This response is partially recapitulated by the inert viscous polymer polyvinylpyrrolidone (PVP), demonstrating that viscosity gradients alone can guide bacterial migration associated with mucosal niche occupation and complement the well-established chemotactic pathways [14]. This study is the only one among the cited works that explicitly interrogates the crosstalk between multiple taxis modalities, dissecting viscotaxis from canonical chemotaxis by comparing cellular responses to mucin and inert viscous polymers. The fact that mucin elicits a stronger migratory response than PVP at equivalent viscosities indicates that viscotaxis and chemotaxis act synergistically in physiological settings. Chemical cues likely amplify the directional bias established by the physical viscosity gradients. This synergy between chemo- and viscotaxis may represent a general principle in mucosal colonization, where both biochemical attractants and physical gradients guide pathogens to their preferred niches.

### 5.5. Steric Exclusion-Based Viscotaxis in Embryonic Tissue Models

Beyond single-cell systems, steric exclusion mechanisms have been proposed theoretically to explain viscosity-directed migration in embryonic tissues. Glycosaminoglycan-rich matrices are proposed to establish local viscosity gradients within the embryonic matrix that guide the migration of cells and neurites. Cell surface polysaccharide coats further fine-tune this process by directly associating matrix viscosity with developmental cell patterning and morphogenesis. This steric exclusion model offers a compelling alternative to the hydrodynamic principles that govern bacterial and single-cell viscotaxis. Rather than relying on propulsion-generated forces, this mechanism operates through the physical constraints imposed by the matrix on cell-surface macromolecules. The finding that matrix viscosity regulates cell adhesion strength through steric exclusion further connects viscosity-directed migration to the core effector of haptotaxis. This indicates that viscosity and adhesion ligand gradients are not independent inputs but converge on shared molecular players, specifically, cell-surface proteoglycans [23]. This convergence raises the intriguing possibility that, at least in certain developmental contexts, viscotaxis and haptotaxis may represent two facets of a shared mechanochemical mechanism, wherein the physical and adhesive properties of the matrix are inextricably linked, though this hypothesis awaits direct experimental testing.

### 5.6. Evolutionary and Technical Insights Across Viscotaxis Model Systems

Collectively, these findings spanning first-principle hydrodynamics, cellular structural determinants, physiological roles, and developmental functions systematically delineate the diverse mechanisms underlying viscotactic migration along gradients of extracellular fluid viscosity or matrix loss modulus. These findings provide cross-scale theoretical and experimental support for the mechanical regulation of directed cell motility in the microenvironment.

A primary observation emerging from this literature is that the heavy reliance on bacterial and microbial systems in early viscotaxis research is not a limitation but a strategic necessity for three complementary reasons. First, the locomotion of bacteria and flagellated microswimmers is driven solely by hydrodynamic interactions, without confounding variables, such as focal adhesion dynamics or intracellular signaling networks, rendering them ideal minimal systems to establish the fundamental physical rules governing viscotaxis through well-validated theoretical frameworks [38]. Second, microorganisms naturally inhabit niches with steep viscosity gradients, including intestinal mucus layers and tissue interstitial fluids, where viscotaxis facilitates niche colonization and pathogenic invasion, highlighting its physiological and ecological relevance [10,24]. Third, assessing viscotaxis in somatic eukaryotic cells requires the rigorous decoupling of matrix loss-modulus gradients from substrate stiffness and adhesion-ligand gradients. This technical barrier remained largely unaddressed until the development of engineered viscoelastic biomaterials [2,19]. This may explain why definitive demonstrations of viscotaxis in mammalian cells have emerged significantly later than in microbial systems and to date remain confined to a limited number of experimental models. This research trajectory reflects the broader paradigm of directed migration studies, in which core physical mechanisms are first validated in simple, well-controlled systems before being extended to complex, multicellular contexts. This bottom-up approach, from microbes to mammals and hydrodynamics to adhesion, reflects the intellectual evolution of the field and highlights the continuity between the physical and biological mechanisms underlying directed migration. Therefore, viscotaxis research illustrates how the study of fundamental physics in simple organisms can illuminate principles that remain operative, although obscured by additional layers of complexity in higher organisms [30]. In line with this trajectory, recent work in mammalian mesenchymal stem cells demonstrates that the same viscosity-sensing machinery operates across substrates of different stiffness and stress relaxation to regulate lineage specification and mechanical memory, extending viscosity sensing from the control of cell motility to the control of cell fate [9].

## 6. Challenges and Perspectives

Despite the advances summarized above, viscotaxis remains a nascent field marked by fundamental knowledge gaps, methodological bottlenecks, and unresolved conceptual debates. Conceptually, the literature still tends to conflate extracellular fluid viscosity, matrix loss modulus, and stress relaxation, and evidence obtained under uniform viscosity is frequently extrapolated—without direct validation—to gradient conditions. Methodologically, generating stable, steep, and quantifiable viscosity gradients that are decoupled from stiffness and adhesion-ligand gradients remains difficult; the molecular identity of the primary viscosity sensor is unknown; and direct in vivo evidence in native mammalian tissues is essentially absent. The following subsections outline these limitations in detail and discuss experimental strategies that may resolve them.

### 6.1. Gradient-Specific Mechanosensing Versus Global Viscosity Perception

A central unresolved question concerns the molecular logic that distinguishes viscosity gradients from uniform viscous environments. While the ARP2/3-NHE1-TRPV4-RhoA/ROCK cascade defines a mechanotransductive axis responsive to elevated extracellular viscosity, it remains unclear whether cells deploy dedicated sensors to detect spatial variations in fluid viscosity or loss modulus, as opposed to uniform viscosity changes [7]. Membrane ruffling and lamellipodial actin dynamics have been implicated in general viscosity mechanosensing, but gradient-specific adaptation, such as asymmetric receptor distribution or polarized ion channel activation across the cell body, has yet to be demonstrated [4]. Addressing this gap will require integrated approaches combining microfluidically generated stable viscosity gradients with high-content live-cell imaging of signaling reporters (e.g., FRET-based RhoA, calcium-sensitive GCaMP, and membrane tension probes) at single-cell resolution [39]. Furthermore, genetic and pharmacological dissection of candidate mechanosensors, including TRPV4, PIEZO1, and other mechanosensitive ion channels, under gradient versus uniform viscosity conditions could reveal pathway-specific contributions.

### 6.2. In Vivo Validation and Multi-Cue Integration

The physiological relevance of viscotaxis remains largely inferred from in vitro reductionist models, with direct in vivo evidence scarce. Native tissues present superimposed gradients of fluid viscosity, matrix stiffness and loss modulus, adhesion ligands, and soluble chemoattractants, making it difficult to attribute migratory behaviors to viscosity alone [30]. To overcome this, advanced intravital imaging in transparent organisms (e.g., zebrafish) or optically accessible tissues could directly track cell trajectories relative to endogenous viscosity gradients mapped by fluorescent microrheology or viscosity-sensitive probes. In parallel, engineered biomaterials with independently tunable stiffness and loss modulus gradients offer defined environments to systematically map how loss-modulus gradients compete with or augment durotactic, haptotactic, and chemotactic cues [19]. Quantitative models incorporating gradient alignment and steepness will be instrumental in predicting migratory outcomes [18].

### 6.3. Three-Dimensional Tissue Architectures and Confinement

The near-exclusive reliance on two-dimensional surfaces and microfluidic channels represents a critical methodological bottleneck. Three-dimensional tissues impose distinct biophysical constraints—including matrix remodeling, steric hindrance, confinement, and hydraulic resistance—that fundamentally alter migratory dynamics. Confinement itself is a multifaceted input: beyond hydraulic resistance, which induces phenotypic transitions that optimize force output in confined channels [27,28], confined migration involves deformation of the nucleus—the largest and stiffest organelle—together with adhesion-independent force transmission, ion-channel-mediated volume regulation, and metabolic reprogramming between glycolysis and oxidative phosphorylation to meet the energetic cost of migration in dense microenvironments [26]. Systematic investigation of viscotaxis in three-dimensional viscoelastic matrices with controlled loss-modulus gradients is currently lacking. In this context, three-dimensional in vitro culture models—cells embedded within viscoelastic hydrogels whose loss modulus can be tuned independently of stiffness, organoids, and ex vivo tissue slices preserving native viscoelasticity—offer intermediate-complexity systems to validate viscotactic responses prior to in vivo studies. Biomaterial scaffolds are particularly promising tools in this regard: their pore architecture, hydraulic resistance, and viscoelasticity can be engineered to impose defined viscosity and loss-modulus environments, and emerging techniques, such as two-photon lithography for fabricating three-dimensional viscoelastic scaffolds and magnetic microrheology for mapping local viscosity in opaque matrices, could establish how scaffold properties shape viscotaxis and enable its exploitation in tissue engineering [19].

### 6.4. Translational Opportunities and Therapeutic Targeting

From a therapeutic perspective, the viscotactic signaling cascade presents tractable nodes for pharmacological intervention, and pharmacological inhibitors of its individual components—including the ARP2/3 inhibitor CK666, the NHE1 inhibitor cariporide, and TRPV4 antagonists—are already available as experimental tools [9]. Targeting the ARP2/3-NHE1-TRPV4 axis could modulate metastatic dissemination in cancers where elevated interstitial fluid viscosity promotes invasion [7]. However, such strategies require careful consideration of context specificity, as these pathways also mediate essential physiological functions in immune cells and epithelial homeostasis. Advanced drug delivery systems—for instance, viscosity-responsive nanocarriers that release inhibitors specifically in regions of elevated viscosity or loss modulus—could enhance therapeutic precision. Collectively, these directions could ultimately establish viscotaxis as a meaningful component of the mechanobiology toolkit, provided that its existence and physiological relevance are convincingly validated in future studies.

## 7. Conclusions

Viscotaxis—directed cell migration along gradients of extracellular fluid viscosity or matrix loss modulus—has evolved from a 1970s behavioral observation in bacteria into a candidate mechanotaxis modality with proposed roles in microbial niche colonization, embryonic morphogenesis, stem-cell fate specification, and cancer invasion. In this review, we have defined viscotaxis and distinguished it from related mechanical quantities, summarized its physiological and pathological relevance, delineated its proposed mechanosensory cascade, examined its potential crosstalk with durotaxis, haptotaxis, chemotaxis, electrotaxis, and phototaxis, and compared the distinct hydrodynamic and steric-exclusion mechanisms that operate across biological systems. At the same time, the field faces clear limitations: much of the mechanistic evidence derives from uniform viscosity elevation rather than true gradients; the identity of the primary viscosity sensor remains unknown; several proposed crosstalk mechanisms—particularly those involving haptotaxis and electrotaxis—rest on theoretical or indirect evidence; and direct in vivo validation in mammalian tissues is lacking. Future progress will depend on engineered viscoelastic biomaterials and microfluidic platforms that generate decoupled, quantifiable gradients; on three-dimensional, organoid, and scaffold-based models that recapitulate native confinement; on intravital imaging combined with viscosity mapping; and on the genetic and pharmacological dissection of candidate mechanosensors under gradient conditions. If these challenges are met, viscotaxis could be established as a core principle of cellular mechanobiology and exploited therapeutically—for example, by targeting the ARP2/3-NHE1-TRPV4-RhoA/ROCK axis to limit metastatic dissemination or by designing viscosity-programmed biomaterial scaffolds that direct cell migration and fate in tissue regeneration.

Taken together, the challenges and perspectives of this nascent field are closely intertwined: the key challenges—demonstrating bona fide viscotaxis under true gradient conditions, identifying the primary sensors of fluid viscosity and loss modulus, and validating the phenomenon in vivo—are precisely the directions in which the field now stands to gain the most. How the community addresses these challenges will determine whether viscotaxis matures from an emerging concept into an established principle of cellular mechanobiology.

## Data Availability

No new data were created or analyzed in this study. Data sharing is not applicable to this article.
